# Intramuscular Botulinum Neurotoxin Serotypes E and A Elicit Distinct Effects on SNAP25 Protein Fragments, Muscular Histology, Spread and Neuronal Transport: An Integrated Histology-Based Study in the Rat

**DOI:** 10.3390/toxins16050225

**Published:** 2024-05-12

**Authors:** Vincent Martin, Denis Carre, Heloise Bilbault, Sebastien Oster, Lorenzo Limana, Florian Sebal, Christine Favre-Guilmard, Mikhail Kalinichev, Christian Leveque, Virginie Boulifard, Catherine George, Stephane Lezmi

**Affiliations:** 1 Ipsen Innovation, 91940 Les Ulis, France; 2 Aix-Marseille University, INSERM, DyNaMo U1325, 13009 Marseille, France

**Keywords:** botulinum neurotoxin type A, botulinum neurotoxin type E, SNAP25, immunohistochemistry, histopathology, muscle, spinal cord, rat

## Abstract

Botulinum neurotoxins E (BoNT/E) and A (BoNT/A) act by cleaving Synaptosome-Associated Protein 25 (SNAP25) at two different C-terminal sites, but they display very distinct durations of action, BoNT/E being short acting and BoNT/A long acting. We investigated the duration of action, spread and neuronal transport of BoNT/E (6.5 ng/kg) and BoNT/A (125 pg/kg) after single intramuscular administrations of high equivalent efficacious doses, in rats, over a 30- or 75-day periods, respectively. To achieve this, we used (i) digit abduction score assay, (ii) immunohistochemistry for SNAP25 (N-ter part; SNAP25^N-ter^ and C-ter part; SNAP25^C-ter^) and its cleavage sites (cleaved SNAP25; c-SNAP25^E^ and c-SNAP25^A^) and (iii) muscular changes in histopathology evaluation. Combined in vivo observation and immunohistochemistry analysis revealed that, compared to BoNT/A, BoNT/E induces minimal muscular changes, possesses a lower duration of action, a reduced ability to spread and a decreased capacity to be transported to the lumbar spinal cord. Interestingly, SNAP25^C-ter^ completely disappeared for both toxins during the peak of efficacy, suggesting that the persistence of toxin effects is driven by the persistence of proteases in tissues. These data unveil some new molecular mechanisms of action of the short-acting BoNT/E and long-acting BoNT/A, and reinforce their overall safety profiles.

## 1. Introduction

The existence of eight different serotypes of botulinum neurotoxin (BoNT) has been reported, from BoNT/A to BoNT/G [1], and, more recently, BoNT/X [2]. While their intracellular targets, receptors, potency and toxicity vary substantially between BoNT serotypes and subtypes, their common feature remains the neurotransmitter release inhibition from nerve terminals through the prevention of SNARE-mediated exocytosis. At the neuromuscular junction, this action can result in flaccid paralysis, and this property has been used over the past decades to treat various neuromuscular conditions [3,4]. Their remarkable safety profile, diversity and versatility for other target tissues have been increasing their interest in curing conditions beyond the neuromuscular junction [5].

BoNT/A is the most used in clinical practice, as well as the most studied in fundamental research. Regarding its action on SNAREs, this serotype does not target syntaxin or synaptobrevin proteins, but acts on Synaptosome-Associated Protein 25 (SNAP25) by cleaving and removing 9 C-terminal amino acid residues to this SNARE protein, and displays a very long duration of action of around 4 to 6 months in human [6,7]. After BoNT/A intramuscular injection, passive diffusion of the toxins is expected within the injected muscle to achieve targeted therapeutic efficacy. However, local (in very close neighboring muscles) and distant (to remote muscles) spread can sometimes be observed at high dose ranges, leading to various exaggerated effects. In addition, neuronal transport using axonal retrograde transport was also evidenced for this serotype in animal studies following peripheral treatment, leading to the presence of SNAP25 cleavage in related central motor nuclei (see [5] for a review of BoNT spread and neuronal transport).

BoNT/E is another serotype that also cleaves SNAP25 but removes a fragment of 26 C-terminal amino acid residues. Surprisingly, and despite a very close mechanism of action, BoNT/E exhibits a strikingly much shorter duration of action compared to BoNT/A with an inhibitory effect of only a few weeks in human muscles [8,9]. Many hypotheses have been formulated to explain such differences in the duration of action between the two serotypes, including differential subcellular localization, degradation or persistence of cleaved products and proteases, but without a clear consensus [10,11,12]. Moreover, neuronal transport of BoNT/E has been much less studied than the one of BoNT/A, mainly using in vitro settings [13]. The same conclusion can be reached regarding the investigation of BoNT/E’s ability to spread to local and distant muscular levels, with scarce research on the topic [14]. Finally, in vivo head-to-head comparisons of the biological effects of both BoNT serotypes are rarely described [15].

The aim of the present study was thus to investigate the biological activity of BoNT/E and BoNT/A to better understand their common features as well as their differences.

## 2. Results

### 2.1. Duration of Action and Local Spread of BoNT/E and BoNT/A

**Figure 1:**
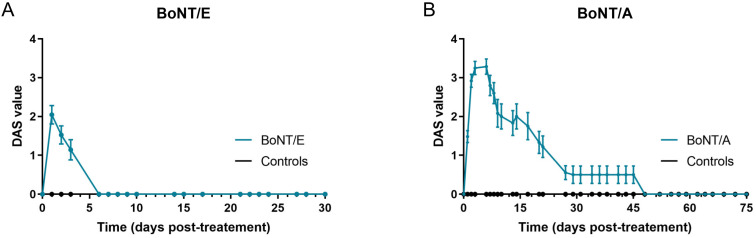
Intramuscular injection of BoNT/E and BoNT/A in rat gastrocnemius impacted differently DAS assay. (**A**) Quantification of DAS in left hind-paw after intramuscular injection of 6.5 ng/kg BoNT/E or GPB (controls) in left gastrocnemius. (**B**) Quantification of DAS in left hind-paw after intramuscular injection of 125 pg/kg BoNT/A or gelatin phosphate buffer (GPB, controls) in left gastrocnemius. Data are represented as mean ± SEM of *n* = 3 to 24 rats for BoNT/E and BoNT/A, from D1 to D30 and D1 to D75, respectively.

The effect of BoNT/E and BoNT/A was first assessed on in vivo rat muscle function using the DAS assay, which is routinely used to evaluate BoNT efficacy in rodents by following the inhibition of hindlimb finger abduction [16]. Extensor Digitorum Longus (EDL) is the muscle involved in the spontaneous reflex controlling this movement [17]. However, in the present conditions, BoNT/E and BoNT/A were injected into a neighboring muscle, the gastrocnemius. An effect on DAS will therefore be indicative of a local spread of the toxins from the injected gastrocnemius to adjacent EDL muscle. For BoNT/E, the peak of abduction inhibition was observed 24 h post-administration (mean DAS value = 2) and went back to normal (DAS = 0) from Day 6 (D6) (Figure 1A). The inhibitory effect of BoNT/A was higher in terms of amplitude and kinetics (Figure 1B), indicating a greater local spread and duration of action. It started at D1, was maximal at D3 to D6 (DAS = 3.25 to 3.3) and progressively decreased up to D50. Of note, no body weight loss or any other sign of BoNT toxicity was observed in any group throughout the study.

### 2.2. Quantification of SNAP25 Cleavage and Fragments in Muscles

The cellular effects of BoNT/E and BoNT/A were then evaluated using immunohistochemistry (IHC) by analyzing the cleavage of their target SNAP25 in rat muscles, at different time points.

**Figure 2:**
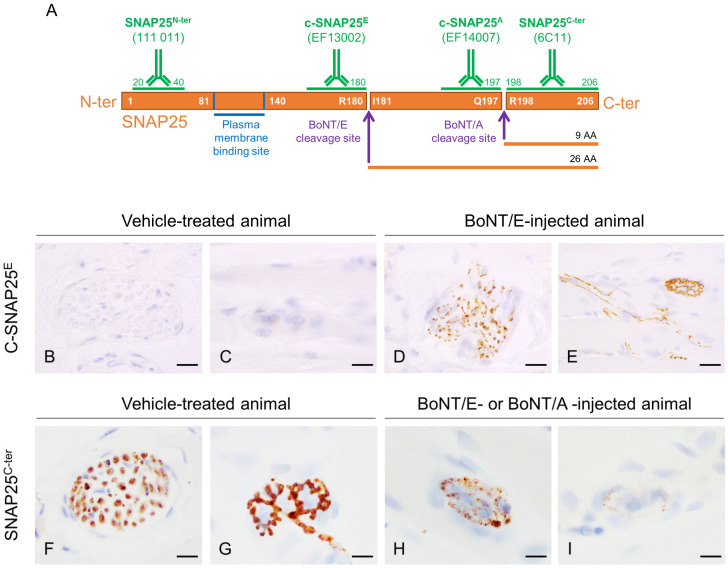
Representation of rat SNAP25 protein, cleavage sites of BoNT/E and BoNT/A and regions targeted by antibodies used in this work, and representative images of c-SNAP25^E^ and SNAP25^C-ter^ staining in rat muscle. (**A**) BoNT/E and BoNT/A cleave SNAP25 at C-ter part, at R180-I181 and Q197-R198, respectively. Antibody 111 011 recognizes both noncleaved and cleaved forms of SNAP25 N-ter part (SNAP25^N-ter^), EF14007 and EF13002 specifically recognize BoNT/E- and BoNT/A-cleaved SNAP25 (c-SNAP25^E^, c-SNAP25^A^), respectively, and 6C11 recognizes SNAP25 C-ter part located right after BoNT/A and BoNT/E cleavage sites (SNAP25^C-ter^). In vehicle-treated rat muscle, c-SNAP25^E^ was absent in nerve bundle (**B**) and at neuromuscular junction (**C**), but was observed in BoNT/E-treated rat muscle (**D**,**E**). SNAP25^C-ter^ was present in nerve bundle (**F**) and at neuromuscular junction (**G**) in vehicle-treated rat muscle, but progressively disappeared in BoNT/E- and BoNT/A-treated rat muscle (**H**,**I**). Scale bars; 20 µm, except for (**D**,**E**); 50 µm.

As expected and already seen with BoNT/A [18], c-SNAP25^E^ staining was observed only in BoNT/E-treated animals and not in control rats, at neuromuscular junctions and intramuscular nerve bundles (Figure 2B–E).

**Figure 3:**
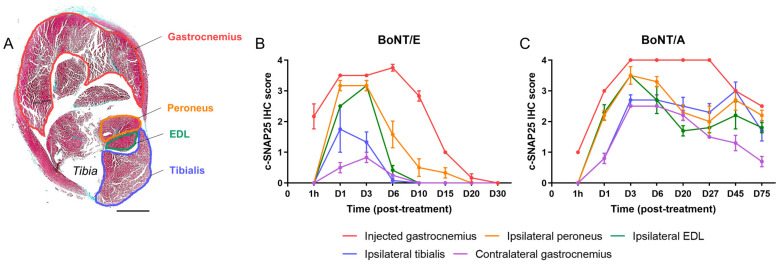
Kinetics and amplitude of c-SNAP25^E^ and c-SNAP25^A^ staining in injected, adjacent and contralateral muscles were different after BoNT/E or BoNT/A administration in rat gastrocnemius. (**A**) Representation of a vehicle-treated rat hindleg cross-sectioned at the level of the gastrocnemius. Injected (gastrocnemius) and adjacent (ipsilateral peroneus, EDL and tibialis) muscles are identified. Scale bar; 4 mm. (**B**) Quantification of c-SNAP25^E^ staining in injected, adjacent and contralateral muscles, after intramuscular injection of 6.5 ng/kg BoNT/E in left gastrocnemius. (**C**) Quantification of c-SNAP25^A^ staining in injected, adjacent and contralateral muscles, after intramuscular injection of 125 pg/kg BoNT/A in left gastrocnemius. Data are represented as mean ± SEM of *n* = 3 rats per time point. Vehicle-treated animals did not show any c-SNAP25^E^ or c-SNAP25^A^ staining throughout this study and were not represented.

In the injected gastrocnemius (Figure 3B,C), detection of c-SNAP25 was noted from 1 h after injection and was higher with BoNT/E than BoNT/A for this time point, indicating a faster action for the former serotype. The maximum amount of c-SNAP25 was equivalent between the two serotypes and observed at D6 for BoNT/E (IHC score = 3.75) and between D3 and D27 for BoNT/A (IHC score = 4). c-SNAP25^E^ staining then rapidly decreased with only traces found at D20 and no more at D30, while c-SNAP25^A^ staining remained moderately present up to the end of the study, suggesting a prolonged biological effect (D75, IHC score = 2.5). In the ipsilateral muscles (Figure 3B,C), levels of c-SNAP25 were overall lower than in the injected gastrocnemius for both toxins and higher with BoNT/A compared to BoNT/E (max IHC scores = 1.75 to 3.2 for BoNT/E and 2.7 to 3.5 for BoNT/A). This result is also indicative of a higher local spread for A serotype. Interestingly, a “diffusion gradient” was observed, as the closer the muscles were located to the injected gastrocnemius, the higher the levels of c-SNAP25, and its presence also tended to last longer with both toxins (peroneus > EDL > tibialis). In these muscles, c-SNAP25 staining kinetics overall followed the pattern of the injected muscle, with a delayed onset. Intriguingly, a “rebound effect” (increase in c-SNAP25^A^ score) was noted with BoNT/A in adjacent muscles at D45 only, which was however not present in the paired contralateral muscles (data not shown). In the contralateral gastrocnemius (Figure 3B,C), only traces of c-SNAP25^E^ were transiently observed with BoNT/E (D1 to D6, IHC scores < 1), while for BoNT/A c-SNAP25^A^ staining evolution was similar to the one observed in the ipsilateral tibialis (max IHC score = 2.5 at D3) with a faster but not complete decrease over time. Distant spread was therefore stronger with BoNT/A than BoNT/E. Interestingly, similar c-SNAP25^A^ levels and patterns were also observed in more distant muscles such as the diaphragm and triceps brachialis (forelimb), compared to what was obtained in the contralateral gastrocnemius with BoNT/A (internal data).

**Figure 4:**
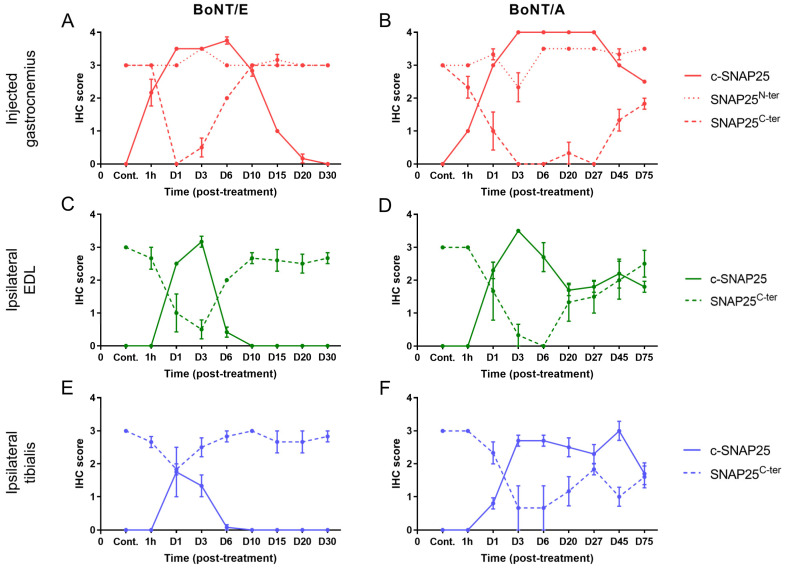
Evolution of SNAP25^C-ter^ staining was inversely proportional to the one of c-SNAP25^E^ and c-SNAP25^A^ in injected and adjacent muscles after BoNT/E or BoNT/A administration in rat gastrocnemius. Quantification of c-SNAP25^E^, SNAP25^N-ter^ and SNAP25^C-ter^ in injected gastrocnemius (**A**), ipsilateral EDL (**C**) and ipsilateral tibialis (**E**) after intramuscular injection of 6.5 ng/kg BoNT/E in left gastrocnemius. Quantification of c-SNAP25^A^, SNAP25^N-ter^ and SNAP25^C-ter^ in injected gastrocnemius (**B**), ipsilateral EDL (**D**) and ipsilateral tibialis (**F**) after intramuscular injection of 125 pg/kg BoNT/A in left gastrocnemius. Data are represented as mean ± SEM of *n* = 3 (BoNT/E or BoNT/A) rats per time point or *n* = 5 (vehicle). Cont.; control (vehicle-treated) animals.

To study more deeply the action of BoNT/E and BoNT/A on SNAP25 protein, the fate of its fragments SNAP25^N-ter^ and SNAP25^C-ter^ was assessed using IHC. For the first one, the antibody used allowed the detection of both the cleaved and non-cleaved forms of SNAP25, as its epitope was located at the N-ter part of the cleavage and membrane binding sites (Figure 2A). In the injected gastrocnemius of vehicle-treated animals, levels of SNAP25^N-ter^ were high, as expected (Figure 4A,B). After treatment with BoNT/E or BoNT/A, it was globally comparable to controls and stable throughout the study with minor changes (IHC scores = 2.5 to 3.5). A slight peak at 3.5 was indeed noted at D3 for BoNT/E, and a more sustained increase from D6 to D75 for BoNT/A. For SNAP25^C-ter^, the antibody-targeted the last region of SNAP25, at the C-ter part of the cleavage and membrane binding sites. This fragment is released after BoNT/E or BoNT/A protease activity on SNAP25. Consequently, SNAP25^C-ter^ staining was strongly observed at nerve bundles and neuromuscular junctions of vehicle-treated rats (Figure 2F,G), but rapidly disappeared after toxin administration in BoNT/E- or BoNT/A-treated animals (Figure 2H,I). Strikingly, in the injected muscle, SNAP25^C-ter^ staining levels were found to be rigorously inversely proportional to the ones of c-SNAP25^E^ and c-SNAP25^A^, in terms of kinetics pattern and amplitude of score. The same observation was also made for both toxins in the ipsilateral EDL and tibialis (Figure 4C–F).

### 2.3. Quantification of Myofiber Atrophy and Histopathological Changes in Muscles

The effect of BoNT/E and BoNT/A on myofibers was then evaluated by assessing the evolution of their size and the presence of histopathological changes in rat muscles, at different time points.

**Figure 5:**
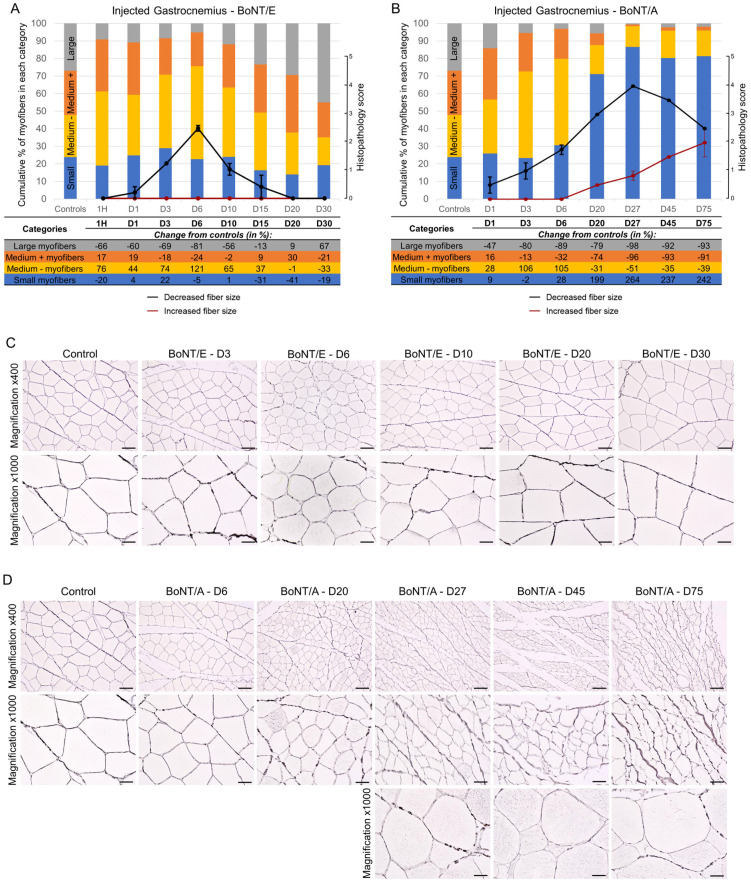
Intramuscular injection of BoNT/E or BoNT/A in rat gastrocnemius impacted myofiber sizes differently. (**A**) Quantification of myofiber size using image analysis (histograms and table) or histopathology (line graphs) in injected gastrocnemius after intramuscular injection of 6.5 ng/kg BoNT/E. (**B**) Quantification of myofiber size using image analysis (histograms and table) or histopathology (line graphs) in injected gastrocnemius after intramuscular injection of 125 pg/kg BoNT/A. Data are represented as the mean of the proportion of myofibers in each size category of *n* = 3 (BoNT/E or BoNT/A) rats per time point or *n* = 5 (vehicle) (histograms), or as mean ± SEM of *n* = 3 (BoNT/E or BoNT/A) rats per time point (line graphs). Control; vehicle-treated animals. (**C**) Representative images of reticulin staining allowing the segmentation of myofibers in injected gastrocnemius after intramuscular injection of 6.5 ng/kg BoNT/E. (**D**) Representative images of reticulin staining allowing the segmentation of myofibers in injected gastrocnemius after intramuscular injection of 125 pg/kg BoNT/A. Scale bars; 50 µm for Magnification ×400 and 20 µm for Magnification ×1000.

For all animals, the size of injected gastrocnemius myofibers was quantified using image analysis, then sorted by categories, and the proportion of myofibers in each size category was followed over time. For BoNT/E (Figure 5A; columns and table, and Figure 5C), the myofiber atrophy was observed by a decrease in the proportion of “large” myofibers was observed from D1 to D15, with a peak at D6 (−81% vs. controls) followed by a normalization. This was correlated to an increase in the proportion of “medium -” but not “small” myofibers. In contrast, myofiber atrophy was much more pronounced and lasted much longer with BoNT/A (Figure 5B; columns and table, and Figure 5D). A substantial increase in the proportion of “small” myofibers was seen from D6 and remained up to D75, with a peak at D27 (+264% vs. controls). The proportion of myofibers in the other categories was globally inversely proportional to the latter one. Myofiber size quantification results were confirmed by histological analysis of the samples (Figure 5A,B; line graphs). A mild to moderate decreased fiber size was observed for BoNT/E while this parameter was marked for BoNT/A (histopathological max score = 2.5 at D6 and 4 at D27, respectively). Interestingly, for some myofibers, an increased fiber size was noted from D20 and progressively intensified up to D75, for BoNT/A only. This phenomenon was mostly observed at the internal part of the muscle, while atrophied myofibers were principally noted at the external and peripheral part of the muscle at the later time points.

**Figure 6:**
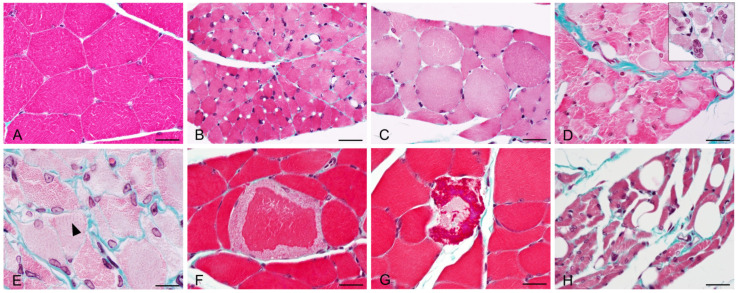
Representative images of histopathological lesions observed using Masson’s Trichrome staining in injected gastrocnemius after intramuscular injection of 125 pg/kg BoNT/A. (**A**) Normal aspect of myofibers in vehicle-treated rat. (**B**) Moderate myofiber atrophy (BoNT/A, D20). (**C**) Increased myofiber size with rounded aspect (BoNT/A, D75). (**D**) Myofiber cytoplasmic pallor, most likely due to myofilament autophagy. Insert: central nuclei in atrophied myofibers (BoNT/A, D20). (**E**) Reappearance of cytoplasmic striations (black arrowhead, BoNT/A, D27). (**F**,**G**) Myofiber degeneration with cytoplasmic fragmentation (BoNT/A, D20). (**H**) Myofiber atrophy with fatty changes; cytoplasmic-clear vacuoles, most likely lipidic (BoNT/A, D75). Scale bars; 20 µm.

Besides myofiber atrophy, other histopathological changes were only found in the injected muscle of BoNT/A-treated animals (Figure 6 and Table 1), no observation was made for the BoNT/E group. Only minimal to moderate toxicological findings were noted, such as myofiber cytoplasmic pallor, myofibers with central nuclei (sign of regeneration), myofibers with fatty changes (most likely lipidic vacuoles) and rare signs of myofiber degeneration/necrosis (maximum two to three myofibers/muscle section when present). Such low severity and frequency findings were expected as already mentioned in the literature, and known to be reversible [19].

### 2.4. Quantification of SNAP25 Cleavage in the Lumbar Spinal Cord

**Figure 7:**
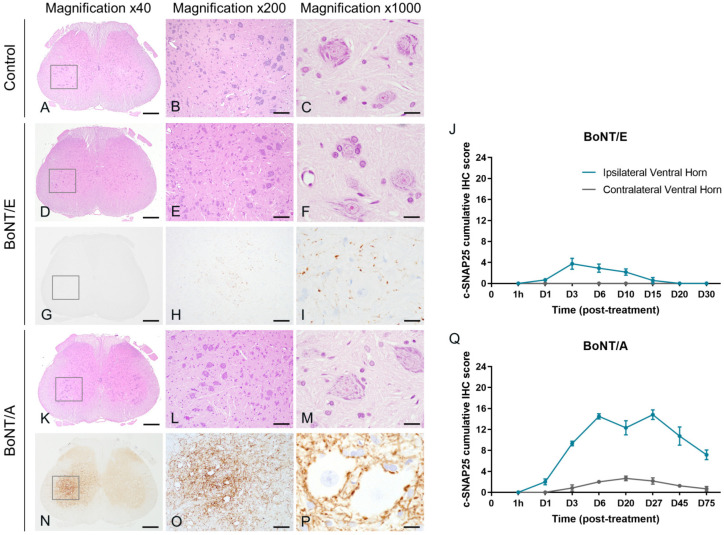
Intramuscular injection of BoNT/E or BoNT/A in rat gastrocnemius triggered distinct SNAP25 cleavage in the lumbar spinal cord without histopathological changes. Representative images of H&E staining on lumbar spinal cord sections from rats injected intramuscularly in the left gastrocnemius with vehicle (**A**–**C**), 6.5 ng/kg BoNT/E (**D**–**F**) or 125 pg/kg BoNT/A (**K**–**M**). Representative images of SNAP25 cleavage and its quantification in lumbar spinal cord sections from rats injected intramuscularly in the left gastrocnemius with 6.5 ng/kg BoNT/E (**G**–**J**) or 125 pg/kg BoNT/A (**N**–**Q**). Data are represented as mean ± SEM of *n* = 3 rats per time point. Vehicle-treated animals did not show any c-SNAP25^E^ or c-SNAP25^A^ staining throughout the study and were not represented. Scale bars; 500 µm for Magnification ×40, 100 µm for Magnification ×200 and 20 µm for Magnification ×1000.

In the lumbar spinal cord, and as expected from previous work [20], no c-SNAP25 staining was observed in vehicle-treated animals. However, the presence of c-SNAP25 was noted with both toxins mainly in the ipsilateral part of the ventral horn close to the large motoneurons, indicating neuronal transport (Figure 7G–I,N–P). For both toxins, the staining was exclusively extra-neuronal, with a synaptic and dendritic-like pattern. No evidence of SNAP25 cleavage was noted in the soma of motoneurons or other surrounding neurons. C-SNAP25 levels were dramatically lower in the ipsilateral lumbar spinal cord with BoNT/E than with BoNT/A (IHC max scores = 3.75 and 14.8, respectively, Figure 7J,Q), indicating a higher neuronal transport for A serotype. The first detection of c-SNAP25 was noted 24 h post-administration, and the maximum intensity was visualized at D3 for BoNT/E and between D6 to D27 for BoNT/A. From D20, c-SNAP25^E^ staining disappeared but the one of c-SNAP25^A^ remained relatively moderate up to the end of the study (D75). In the contralateral part of the lumbar spinal cord, no SNAP25 cleavage was observed at any time point for BoNT/E, while only traces were noted for BoNT/A, following an ipsilateral pattern (max IHC score = 2.7 at D20). Histopathological analyses on H&E-stained sections (same lumbar spinal cord levels as the ones positive for c-SNAP25) did not show any evidence of morphological changes in any of the rats injected with BoNT/E or BoNT/A at any time of the study (Figure 7A–F,K–M). Similarly, in the same samples, IHC staining for Glial Fibrillary Acidic Protein (GFAP) did not show any evidence of reactive astrogliosis (increased number of astrocytes) or astrocytosis (increased number of glial processes).

## 3. Discussion

Using immunohistochemical and histopathological tools, we were able to compare the mode and the duration of action, the spread and neuronal transport of natural BoNT/E and BoNT/A for a better understanding of their pharmacological activities and safety evaluation.

### 3.1. Study Dose Selection

To elicit and compare significant biological effects and spread without toxic clinical signs, the doses of 6.5 ng/kg (2 ng/rat) for BoNT/E and 125 pg/kg (40 pg/rat) for BoNT/A were selected, which represent ±25 to 28 times their DAS ED_50_ after injection in the peroneus. Such difference in dose range to achieve similar efficacy has already been observed elsewhere [14,15] and could be explained by the lower expression of BoNT/E receptors SV2A and SV2B in rat muscles when BoNT/A also uses the more expressed SV2C protein [21]. This hypothesis suggests that depending on the expression of their receptors in the administered tissue, the efficacy of the various BoNT serotypes can markedly vary. An extensive and exhaustive receptor expression profiling in human tissues would then be of high interest to better appreciate which BoNT serotypes are the most appropriate to treat diseases specific to certain organs.

### 3.2. Duration of Action, SNAP25 Cleavage and Muscle Atrophy

As expected [14,15], BoNT/E demonstrated a faster onset and shorter duration of action compared to BoNT/A in all assays, and the proposed integrated histology-based approach enabled us to compare the kinetics of BoNT/E and BoNT/A in vivo activity with one of their cellular effects. For BoNT/E, the evolution of DAS was rather well correlated to the one of SNAP25 cleavage in EDL, the muscle involved in rodent toe extension [17]. However, some disconnection appeared for BoNT/A, as the spreading was rescued around D20 when c-SNAP25^A^ levels remained moderate up to the end of the study (D75), consistent with previous reports showing persistence of c-SNAP25^A^ at rat neuromuscular junction in similar time frame [22]. This discrepancy could be explained by three hypotheses; (i) the level of SNAP25 cleavage is not elevated enough to elicit muscle paralysis, (ii) nerve sprouting happened and rescued muscle functionality and/or (iii) the presence of c-SNAP25 is not a correlated with cellular presence of proteases. To answer the first point, another study design would have been needed, evaluating for a broad range of doses the correlation between the amount of SNAP25 cleavage and BoNT/A effect in a more sensitive functional test, such as muscle force or compound muscle action potential tests [18]. Regarding the second point, while the present immunohistochemical approach was not intended to study nerve sprouting, we observed in BoNT/A-injected muscle during peak of efficacy (maximal levels of SNAP25 cleavage) growing proportion of myofibers displaying increased size and slight but sustained increase in SNAP25^N-ter^ antibody staining, potentially sign of apparition of new SNAP25 positive neuromuscular junctions. This could have also happened in the EDL muscle and therefore initiated muscle functionality recovery in the DAS. For BoNT/E, the fact that no signs of nerve sprouting were observed supports the theory in [15] that such a phenomenon only happens when blockade of neuromuscular junction occurs over a prolonged period. Moreover, the data from the present work extend it to all BoNT-related tissular consequences, such as histopathological changes. Finally, the third point can be refuted by the IHC analysis of SNAP25^C-ter^ part. The fact that SNAP25^C-ter^ staining at the neuromuscular junction disappeared totally during the maximal effect of the toxins indicates (i) that the SNAP25^C-ter^ fragment unlikely interferes with SNARE machinery and is not per se involved in BoNT inhibitory action, and (ii) that all former SNAP25 proteins are truncated and newly synthesized ones are continuously cleaved as well. This, and the reappearance of SNAP25^C-ter^ when the toxin activity decreased (reduction in c-SNAP25 staining) clearly suggest that the level of SNAP25 cleavage is directly linked to the cellular presence of active toxins. Persistence of SNAP25 cleavage, and thus BoNT duration of action, is then mediated by the persistence of BoNT proteases at nerve endings [23,24]. Following this idea, improvement in clinical benefit with a higher duration of action could be achieved using strategies aiming at increasing the internalization of BoNT proteases at neuromuscular junctions up to saturation [25] or by decreasing their degradation by the proteasome [12].

### 3.3. Spread and Neuronal Transport

As expected, local and distant spread were observed with BoNT/A. While some studies reported limited spread with indirect evaluation [26], other groups using higher doses and more sensitive methods showed local and distant effects to a similar extent than the present work [27,28,29,30]. Interestingly, a “diffusion gradient” for local spread was observed for both serotypes, which is used under certain clinical circumstances to access higher or deeper target areas [31]. This is in line with previous observations showing that BoNT/A is able to diffuse a few centimeters in muscle [32], but that crossing fascia reduces its spread [33]. Although local spread is known to occur via passive diffusion of BoNT in tissues, the mechanism behind distant spread is still a matter of debate. According to some authors, systemic exposure could happen through the bloodstream and/or neural pathways [28,34] (last point discussed below). Little information was available for BoNT/E safety, which has only been shown to spread remotely with a better therapeutic index than BoNT/A [14], and this is confirmed by the present work. In clinical practice, the lower tissue penetration of BoNT/E would prevent its use for deep muscle targeting. However, this property would be an advantage for more targeted or confined approaches such as strabismus treatment, with reduced local effects and better safety than BoNT/A. Further work is needed to understand BoNT/E’s lower ability to spread, but differences in extracellular stability or capacity to diffuse within tissues and in biological fluids could be involved.

BoNT spread is a topic of particular concern as, in rare cases, it can lead to severe botulism and the absence of an antidote makes difficult its recovery management [35]. Although spread can occur, it seems to be mainly driven by the dose [36,37]. BoNT-based therapies are known to display excellent safety profiles in clinical use when recommendations regarding doses and re-injection frequency are followed [5,38,39,40]. The results of the present study provide a good example of this statement. As discussed earlier, “suprapharmacological” doses were used (>25/28 times the DAS ED_50_) that induced spread, as revealed by the presence of c-SNAP25 in different muscles. However, despite the presence of c-SNAP25 in non-injected muscles, animals were clinically well, demonstrating that moderate and transient SNAP25 cleavage away from injected muscle is not deleterious.

While neuronal transport for BoNT/A is well known [22,34,41], this study is the first to report such a phenomenon in vivo with BoNT/E after peripheral administration, more relevant to clinical practice. The data confirm here that BoNT/E can be axonally transported, but to a lower efficiency compared to BoNT/A [13,42,43]. Autophagosomes have been identified to be the specific carriers for BoNT/A during neuronal transport [44]. Although this must be experimentally confirmed, we can postulate that the lower ability of BoNT/E to undergo such a phenomenon could be due to a potential targeting of other types of cargo less involved in neuronal transport. Many works support the importance of neuronal transport in the antinociceptive activity of BoNT/A [20,45,46]. As such behavior is limited in BoNT/E, it might restraint its therapeutic use for such conditions, maybe explaining the paucity of published data on the subject.

Regarding neuromuscular diseases, the biological significance of SNAP25 cleavage in the spinal cord ventral horn after muscular administration is uncertain. This central action might participate in the mode of action [47,48,49], but nevertheless the present study clearly suggests that BoNT activity at the spinal cord level is biologically safe, as no histopathological tissular reactions were observed. Some authors hypothesize that the distant spread of BoNT, or at least some serotypes, occurs through neuronal transport to the spinal cord and then to contralateral muscles [27]. The present work brings some new mechanistic elements that refute this theory. In the contralateral part of the lumbar spinal cord, only traces of c-SNAP25 staining were observed for BoNT/A and absolutely nothing for BoNT/E. This emphasizes the fact that if BoNT/A can enter second-order neurons [50,51], it does not diffuse extensively in central tissues [52]. The disconnection between SNAP25 cleavage kinetics in the contralateral muscle (maximal at D3) and spinal cord (moderate at D3, maximal from D6) is another strong argument.

Together, these spread and neuronal transport results emphasize the fact that, beyond pharmacological properties, the various BoNT serotypes also greatly differ in terms of safety and neuronal fate. Recombinant technology is now used to produce BoNT chimeras combining different parts of BoNT serotypes [53,54,55]. If this offers many interesting possibilities, it also comes with some challenges, one being the definition of optimal dose, as depicted in the present study where the two serotypes displayed a very different range of potency and toxicity. In our example, BoNT/A being used at the same dose as BoNT/E would be very toxic, and BoNT/E used as the dose of BoNT/A would not be efficacious. The compatibility of BoNT serotypes needs to be experimentally assessed to guarantee a good balance between efficacy and safety. 

## 4. Conclusions

In conclusion, this study unveils some new molecular mechanisms of action of the short-acting type E and long-acting type A botulinum neurotoxins. The results highlight the striking differences in biological features that can exist between BoNT serotypes. With the emergence of BoNT engineering, it will be possible to produce BoNT chimeras combining the distinct patterns of efficacy, duration of action, spread or neuronal transport of BoNT serotypes. This will help bring to patients new BoNT therapies with enhanced properties, such as longer duration of action, reduced capacity to spread or ability to target specific cell types. Deciphering the biological effects of BoNT serotypes is then key to improving and broaden the use of BoNT therapies in clinical practice.

## 5. Materials and Methods

### 5.1. Animals

Adult male Sprague-Dawley rats (310 ± 15 g at the beginning of treatment) were purchased from Janvier Labs and housed in the Ipsen Innovation animal facilities. Animals were kept on a 12 h light/dark cycle (lights on from 07:00 to 19:00) and maintained in an enriched environment under a constant temperature (22 ± 2 °C) and humidity (55% ± 5%) with food and water available ad libitum. Animals were acclimatized for at least 7 days prior to experimentation. Experimental procedures were reviewed and approved by the Ethics Committees of Ipsen Innovation (C2EA, registration number 32). Studies were performed in full compliance with the ARRIVE guidelines, European Communities Council Directive 2010/63/EU and French National Committee decree 87/848.

### 5.2. BoNT Administration

Research-grade, purified, native BoNT serotypes E and A (BoNT/E, BoNT/A; 150 kDa) were purchased from List Labs, Campbell, California, USA (#141A and #130B, respectively). BoNT/E and BoNT/A were reconstituted in 1 mg/mL bovine serum albumin in Dulbecco’s phosphate-buffered saline (Sigma-Aldrich, Saint-Louis, MO, USA, #A1595) to obtain a 0.1 mg/mL stock solution that was stored at −80 °C as single-use aliquots. Working solutions were prepared with gelatin phosphate buffer (GPB) to obtain the final desired concentrations. Preparation of GPB was done as already described in [18].

Before starting the experiments, animals were weighed, pre-screened for normal digit abduction responses and randomized to obtain a comparable mean body weight in each group. At day 0 (D0), rats were injected under anesthesia (3% isoflurane in oxygen) by intramuscular route in the lateral and medial heads of the left gastrocnemius muscle (10 µL per muscle head, 20 µL in total) with BoNT/E (2 ng/rat = 6.5 ng/kg), BoNT/A (40 pg/rat = 125 pg/kg) or their vehicle (GPB). Dose selection was carefully made based on internal pharmacology and safety data to select for both toxins high but not toxic doses (not leading to body weight loss). 6.5 ng/kg BoNT/E and 125 pg/kg BoNT/A correspond to respectively 25 and 28 times the ED_50_ of each toxin in the rat Digit Abduction Score (DAS), a functional assay widely used to determine BoNT in vivo activity (BoNT/E ED_50_: 265 pg/kg, internal data; BoNT/A ED_50_: 4.4 pg/kg, [16]; administration in the peroneus muscle for both toxins).

### 5.3. Digit Abduction Score (DAS) Evaluation

The DAS was evaluated 5 days per week to assess the local spread of the injected toxins from the gastrocnemius to the Extensor Digitorum Longus (EDL) which controls the abduction of hindlimb fingers. To evaluate the digit abduction response of the left hindlimb, each rat was grasped lightly around the torso and lifted swiftly in the air and scored using a five-point scale (DAS value), from normal reflex/no inhibition (DAS 0) to full inhibition of the reflex (DAS 4), as already described [16].

### 5.4. Immunohistochemistry (IHC) and Histopathology 

Three treated rats were sacrificed at each of the following time points: 1 h, D1 (24 h), D3, D6, D10, D15, D20, D30 post-administration for BoNT/E, or at 1 h, D1, D3, D6, D20, D27, D45, D75 post-administration for BoNT/A. A total of one control (vehicle-treated) rat was sacrificed at each of the following time points: 1 h, D6, D20, D45, D75. Sacrifices were performed by decapitation under deep anesthesia (5% isoflurane in oxygen), according to internal ethical procedures. The injected (left) and contralateral (right) gastrocnemius muscles, the ipsilateral (left) EDL, peroneus and anterior tibialis muscles, and the lumbar spinal cord (multiple cross-sections every 2 mm of L1 to L6 vertebrae) tissues were harvested. Tissues were then fixed in isotonic buffered formalin 10% solution (VWR, Rosny-sous-Bois, France, #9713.5000) for 48 h, embedded in paraffin blocks and histologic slides were prepared.

Different antibodies were used to detect in the tissue samples the different parts and fragments of SNAP25 protein over time using the IHC technique (Figure 2A). To detect c-SNAP25, non-commercial antibodies specific for BoNT/E- or BoNT/A-cleaved form of SNAP25 were used: EF13002 (c-SNAP25^E^; IPSEN) and EF14007 (c-SNAP25^A^; IPSEN), respectively. These antibodies are specific for BoNT/E and BoNT/A cleavage sites and do not recognize non-cleaved SNAP25 protein [18], nor the cleavage site produced by the other toxin. The C-ter part of SNAP25 located right after BoNT/E and BoNT/A cleavage sites was detected by non-commercial antibody 6C11 (SNAP25^C-ter^, INSERM U1325, region 198–206). The N-ter part of SNAP25 was detected by commercial antibody 111 011 (SNAP25^N-ter^, Synaptic Systems, region 20–40), which recognizes both noncleaved and cleaved forms of SNAP25 [18].

The immunohistochemical staining method was performed using a standard avidin-biotin-peroxidase procedure as previously described [20]. After a heat-induced epitope retrieval step, endogenous peroxidases were blocked by 10 min incubation in a 3% H_2_O_2_ solution (Sigma-Aldrich, #H1009). The sections were incubated with the primary antibodies. Sections were then incubated with a biotinylated secondary antibody for 30 min (anti-rabbit or anti-mouse IgG, Vector Laboratories, Newark, CA, USA, #BA-1100 and #BA-2001), followed by a 30-min incubation with an amplification system (avidin–biotin) coupled to horseradish peroxidase (Vector Laboratories, #PK-7100). Finally, sections were incubated for 5 min with a 0.02% diaminobenzidine solution (DAKO, Les Ulis, France, #K346811-2). For counterstaining, hematoxylin (DAKO, #K800821-2) was used, and the slides were finally mounted and evaluated using light microscopy. Slides of positive controls (samples known to display the marker of interest), negative controls (samples known to not display the marker of interest) and isotype controls (replacement of primary antibody by non-specific rabbit or mouse IgG; Vector Laboratories, #I-1000 and #I-2000) were systematically included to guaranty quality and specificity of IHC staining. Classic Hematoxylin and Eosin (H&E) and Masson’s Trichrome stained slides were also prepared for histopathology analyses (LAPV Amboise, France).

For the quantification of the amount of c-SNAP25^E^ and c-SNAP25^A^ in muscles and spinal cord, the same method was used as described in [18,20]. Briefly, in muscles, c-SNAP25 staining was scored from 0 to a maximal score of 4. In the spinal cord, c-SNAP25 staining was scored from 0 to maximal score 4 in the ipsilateral and contralateral ventral horns of the 6 most intensely stained spinal cord sections; a cumulative score (0–24) was then calculated for each animal.

The quantification of the amount of SNAP25^N-ter^ and SNAP25^C-ter^ in muscles at the neuromuscular junction was determined using the following 4-point scale scoring system: 0 (no staining), 1 (minimal staining intensity and density), 2 (moderate staining intensity and density), 3 (strong staining intensity and density) and 4 (very strong staining intensity and density).

All the IHC quantifications were evaluated in a blind manner by at least 2 investigators and reviewed by one Board-Certified Veterinary Pathologist (SL).

### 5.5. Myofiber Atrophy Quantification

To evaluate myofiber atrophy, quantification of myofiber area was performed. For each injected gastrocnemius muscle, a slide of a tissue cross-section taken at the level of the muscle belly was stained with a reticulin contrast kit (Biognost, Zagreb, Croatia, #RE-K-100), allowing identification and segmentation of the myofibers. Stained slides were scanned and the area of each myofiber was automatically quantified using a dedicated and automated computerized method (HALO, Excilone, France). Raw data from the control animals were pooled to allow the determination of 4 categories of myofiber areas using the quartiles method. Briefly, median, quartile 1 (Q1) and quartile 2 (Q2) values were calculated using Prism (GraphPad Software, version 8.3.0). For each animal, the proportion of myofibers in each size category was then calculated based on their area values (“Small” myofibers: <Q1 value, “Medium -” myofibers: between Q1 and median values, “Medium +” myofibers: between median and Q2 values, “Large” myofibers: >Q2 value) and the mean is represented in bar graphs using Excel (Microsoft, version 2018).

Myofiber atrophic and hypertrophic changes as well as other histopathological observations were also quantified using light microscopy using H&E and Masson’s Trichrome slides by a Board-Certified Veterinary Pathologist (SL). The severity of “decreased fiber size”, “increased fiber size” or other observed lesions was quantified using a classical histopathology scoring system used in toxicology (1: minimal, 2: mild, 3: moderate, 4: marked, 5: severe).

### 5.6. Statistical Analysis

Due to the limited number of animals per sacrifice time points (3 for BoNT/E and BoNT/A) and the observational-based scientific approach (immunohistochemistry and histopathology), statistical analyses were considered irrelevant for this work and thus not performed.

## Figures and Tables

**Table 1:** Intramuscular injection of BoNT/A in rat gastrocnemius induced minimal to moderate histopathological changes in the injected muscle. Quantification of histopathological lesions observed in the injected muscle of rats injected intramuscularly in the left gastrocnemius with 125 pg/kg BoNT/A. Data are represented as a mean of *n* = 3 rats per time point. -; not observed, 1; minimal, 2; mild, 3; moderate.

Microscopic Changes	D1	D3	D6	D20	D27	D45	D75
Myofibers, cytoplasmic pallor	-	-	-	3	3	-	-
Myofibers, peripheral cytoplasmic pallor	-	-	-	-	1	1	1
Myofibers, central nuclei	-	-	-	2	2	2	2
Myofibers, degeneration/necrosis	-	-	-	-	1	1	1
Myofibers, lipidic vacuoles (fatty change)	-	-	-	-	-	-	1

## Data Availability

Raw data can be made available on reasonable demand.
